# Fracture Tables. Being a Supplement to the Fracture Tables Published by Dr. Hamilton in the Buffalo Medical Journal, April, 1849

**Published:** 1852-09

**Authors:** J. Boardman

**Affiliations:** Buffalo, N. Y.


ART. II.—*Fracture Tables. Being a Supplement to the Fracture Tables published by Dr. Hamilton in the Buffalo Medical Journal, April*, 1849. Compiled and arranged from DR. HAMILTON'S notes. By J. BOARDMAN, A. B., Buffalo, N. Y.

In the Buffalo Medical Journal for April, 1849, and afterward in pamphlet form, Dr. Frank H. Hamilton published "tables" showing the results of treatment in one hundred and thirty-six cases of fracture for the "purpose of determining the average results of treatment in fractures."

Those tables were compiled from notes made by Dr. Hamilton of such cases as came under his own observation, and which he had the privilege of examining, not drawn exclusively from his own practice, or from the practice of this city, but collected from various sources.

The following additional tables have been compiled from notes of nearly three hundred more cases collected by him since that time, and as in the first tables, no case has been admitted which was known to have been treated by an empiric, or which did not come under surgical treatment.

Dr. Hamilton's measurements have been made with great care, rarely those taken from two points being considered sufficient until proved to be correct by at least one, and often two other measurements. Also in these notes by recording the name of surgeon, name of patient, time, and place of accident, care has been taken to avoid the repetition of any case, and to have them show the true results in cases of fracture.

In the tables the names of *females* are printed in *italics*, *y.* is written for *year*, *m.* for *month*, *w.* for *week*, *u.* for *united*, *n. u.* for *not united*, *in.* for *inch*, *p.* for *perfect*, *ip.* for *imperfect*. A limb is called "perfect" when there is no striking deformity, shortening, or maiming.

| Name of Bones. | Point of Fracture. | Character of Frac. | Name of Patient. | Age then. | Time since it occurred. | Unit'd or not united | Amt. of short'n'ng, | Perfect or imp'rfect |
|---|---|---|---|---|---|---|---|---|
| 170 Ossa nasi, | | compound | T. B. | 17 y. | 14 y. | u. | | ip. |
| 171 " | | " | G. C. | | 4 w. | u. | | ip. |
| 172 " | | simple | C. D. | 11 y. | | u. | | ip. |
| 173 " | | " | N. B. | 25 y. | 2 y. | u. | | ip. |
| 174 " | | " | *H. S.* | 3 w. | 12 y. | u. | | ip. |
| 175 " | | " | *I. A.* | 8 y. | 15 y. | u. | | p. |
| 176 Sept. nasi, | | simple | L. L. | 19 y. | 33 y. | u. | | ip. |
| 177 " | | " | J. B. | 26 y. | 4 y. | u. | | ip. |
| 178 " | | " | J. M. | 28 y. | 3 y. | u. | | ip. |
| 179 Sup. max. | under mal'r | simple | M. P. | 34 y. | 1 y. | u. | | p. |
| 180 Inf. max. | shft.bth.sds. | comp. com. | J. K. | 17 y. | 8 m. | u. | | ip. |
| 181 " | shaft | " | A. G. | 11 y. | 3 y. | u. | | p. |
| 182 " | angle | " | R. B. | 39 w | 7 w. | u. | | ip. |
| 183 " | ang. & shaft | simple | A. M. | 26 y. | 5 w. | n. u. | | ip. |
| 184 " | shaft | " | J. W. S. | 25 y. | 1 y. | u. | | p. |
| 185 " | angle | " | J. McE. | 22 y. | 1 y. | u. | | p. |
| 186 " | " | " | A. V. | 47 y. | 15 y. | u. | | p. |
| 187 " | symp.ment. | " | N. M. | 25 y. | 10 y. | u. | | ip. |
| 188 Clavicle, | out ⅓ | commin. | B. L. D. | | | u. | ½ in. | ip. |
| 189 " | " | simple | S. U. | 47 y. | 11 y. | u. | ¼ in. | ip. |
| 190 " | " | " | W. P. L. | 26 y. | 13 y. | u. | | p. |
| 191 " | " | " | H. S. | 56 y. | 1 y. | u. | ½ in. | ip. |
| 192 " | " | " | E. M. | 14 y. | 31 y. | u. | | p. |
| 193 " | " | " | J. R. | 44 y. | 1 y. | u. | ½ in. | ip. |
| 194 " | " | " | G. W. R. | 48 y. | 2 y. | u. | ½ in. | ip. |
| 195 " | " | " | C. A. W. | 47 y. | 4 y. | u. | ½ in. | ip. |
| 196 " | " | " | *Mrs. —* | 28 y. | 12 y. | u. | ½ in. | ip. |
| 197 " | " | " | *P. T.* | 51 y. | 4 m. | u. | ½ in. | ip. |
| 198 " | " | " | *M. McC.* | 12 y. | 23 y. | u. | ½ in. | ip. |
| 199 " | " | " | *A. A. H.* | 3½ y. | 6 m. | u. | | p. |
| 200 " | " | " | *B. G. C.* | 40 y. | 4 m. | u. | ½ in. | ip. |
| 201 " | middle | " | I. L. | 32 y. | 8 y. | u. | ¼ in. | ip. |

170. The deformity is very conspicuous.

173. Displaced laterally; ulceration of septum nasi, has occurred and still continues; health bad.

174. Nose nearly flat at its middle and upper part.

175. Slight prominence of cartilage at end of nasal bones.

176. Nose perfectly flat.

177. Nose much flattened.

180. Central fragment slightly lifted and displaced.

182. Posterior fragment displaced upward and anteriorly. Necrosis has occurred.

187. Fracture vertical; left side displaced upward and posteriorly.

188. Deformity striking. The usual form of displacement of fractures at this point, i. e. the outer end of inner fragment over rides the inner end of outer fragment.

189. 190. 191. The displacement of same character as No. 188.

192. There was a projection at seat of fracture five years. None now.

193. 194. 195. 196. Displacement of same character as No. 188.

197. The arm was nearly paralyzed, from use of axillary pad.

198. Deformity at seat of fracture for long time great; now very slight.

200. Bent forward at seat of fracture. Arm not as strong.

| Name of Bones. | Point of Fracture. | Character of Frac. | Name of Patient. | Age then. | Time since it occurred. | Unit'd or not united | Amt. of short'ning. | Perfect or imp'rfect |
|---|---|---|---|---|---|---|---|---|
| 202 Clavicle, | middle | simple | R. M. F. | 12 y. | 2 m. | u. | | p. |
| 203 " | " | " | R. M. F. | " | " | u. | | p. |
| 204 " | " | " | J. D. S. | 2 y. | 24 y. | u. | | p. |
| 205 " | " | " | S. McN. | 37 y. | 10 y. | u. | | p. |
| 206 " | " | " | J. C. | 43 y. | " | u. | | p. |
| 207 " | out ⅓ | " | I. E. | 35 y. | 5 y. | u. | ½ in. | ip. |
| 208 " | " | " | M. K. | 50 y. | 1 y. | u. | ½ in. | ip. |
| 209 " | " | " | H. M. | 44 y. | 6 m. | u. | ¾ in. | ip. |
| 210 " | " | " | I. W. | 15 y. | 1 y. | u. | | p. |
| 211 Scapula, | coracoid P. | simple | I. W. | 30 y. | 3 m. | n. u. | | ip. |
| 212 " | below spine | " | W. V. | 54 y. | 1 y | u. | | p. |
| 213 Humerus, | low ⅓ | comp. | J. A. | 12 y. | 10 y. | u. | ¾ in. | ip. |
| 214 " | surg. neck. | simple | M. B. | 23 y. | 2 m. | u. | | p. |
| 215 " | " | " | M. C. | 30 y. | 7 w. | u. | | p. |
| 216 " | up. ¼ | " | J. T. | 54 y. | 9 w. | u. | | p. |
| 217 " | middle | " | J. M. C. | 63 y. | 3 y. | u. | ⅞ in. | ip. |
| 218 " | " | " | E. F. | 18 y. | 25 y. | u. | | p. |
| 219 " | " | " | J. B. | 44 y. | 5 m. | u. | | p. |
| 220 " | " | " | S. | 13 y. | 7 m. | u. | ¼ in. | ip. |
| 221 " | " | " | J. H. S. | 36 y. | 41 y. | u. | | p. |
| 222 " | low ⅓ | " | W. C. | 4 y. | 20 y. | u. | | p. |
| 223 " | " | " | S. McN. | 32 y. | 15 y. | u. | | p. |
| 224 " | " | " | E. S. | 39 y. | 8 y. | u. | | p. |
| 225 " | low ⅛ | " | F. N. | 59 y. | 7 m. | u. | ½ in. | ip. |
| 226 " | " | " | C. F. | 8 y. | 8 m. | u. | ¾ in. | ip. |
| 227 " | " | " | H. A. | 38 y. | 4 m. | n. u. | ½ in. | ip. |
| 228 " | " | " | *E. H.* | 2$\frac{9}{12}$ y. | 3 m. | u. | | p. |

202. 203. Was a *bend* of right and left clavicle.

207. Shoulder half inch lower than other ; use of arm perfect.

210. Inflammation extended to articulation of head of humerus, producing for a long time false anchylosis.

211. Process moves with the head of the humerus.

213 A small piece of the bone was removed. Has false anchylosis of elbow.

214. See No. 283.

217. Seven weeks after accident bone had not united and the fragments were displaced. Same patient as No. 274.

216. Result of fragilitas ossium.

219. Result of lues.

220. Motions of arm nearly perfect.

222. At same time fractured radius and ulna. See No. 271.

223 Cannot straighten arm perfectly.

225. Lower end of upper fragment is in front of the upper end of lower fragment, flexion and extension imperfect.

226. Fracture immediately above condyles. Treated with two right-angle splints, one anteriorly, one posteriorly ; and also two short, lateral splints, for five weeks. At which time, the upper fragment was found to project in front of the upper end of lower fragment ; motion of elbow perfect ; soon, the hand became forcibly flexed upon fore-arm, the first phalanges, upon metacarpal bones, and he lost the power of pronation and supination. The projecting portion, eight months after the fracture, was removed by Dr. Hamilton, but with little or no benefit to patient.

227. Has slight motion of arm, but motion of fore-arm nearly perfect.

229. False anchylosis, lower fragment displaced backward.

| Name of Bones. | Point of Fracture. | Character of Frac. | Name of Patient. | Age then. | Time since it occurred. | Unit'd or not united | Amt. of short'ning. | Perfect or imp'rfect |
|---|---|---|---|---|---|---|---|---|
| 229 Humerus, | low 1-6 | simple | | 9 y. | 2 m. | u. | | ip. |
| 230 " | in condyle | " | P. Y. | 5½y. | 6 m. | u. | | ip. |
| 231 " | " | " | G. B. | 5 y. | 1 y. | u. | | p. |
| 232 " | " | " | B. S. | 12 y. | 38 y. | u. | | p. |
| 233 " | " | " | *A. B. H.* | 43 y. | 6 m. | u. | | ip. |
| 234 " | out. condyle | " | E. R. | inf'cy | ad'lt | u. | | ip. |
| 235 " | " | " | J. R. D. | y. | 11 w | u. | | ip. |
| 236 " | " | " | *R. W.* | 88 y. | 2 m. | u. | | ip. |
| 237 Radius, | lower end | compound | O. P. | 15 y. | 2 y. | u. | | ip. |
| 238 " | lower ⅓ | " | W. H. M. | 16 y. | 15 y. | u. | | p. |
| 239 " | neck | simple | I. R. A. | 25 y. | 10 m | u. | | ip. |
| 240 " | upper ⅓ | " | J. B. | 9 y. | 10 y. | u. | | p. |
| 241 " | middle | " | W. J. | 10 y. | 19 y. | u. | | p. |
| 242 " | " | " | M. O. B. | 45 y. | 8 w. | u. | | ip. |
| 243 " | " | " | D. S. | 21 y. | 50 y. | u. | | p. |
| 244 " | lower ⅓ | " | P. B. | 15 y. | 3 m. | u. | | p. |
| 245 " | " | " | W. L. | 2 9/12 y. | 6 m. | u. | | ip. |
| 246 " | " | " | P. D. | 1⅔ y. | 4 m. | u. | | p. |
| 247 " | " | " | W. H. | 33 y. | 21 y. | u. | | p. |
| 248 " | " | " | H. H. | 14 y. | 24 y. | u. | | p. |
| 249 " | " | " | S. G. | 15 y. | 25 y. | u. | | p. |
| 250 " | " | " | M. W. | 8 y. | 1 y. | u. | | ip. |
| 251 " | " | " | T. B. | 22 y. | 3 m. | u. | | p. |
| 252 " | " | " | *H. D.* | 52 y. | 3 y. | u. | | ip. |
| 253 " | " | " | *I. L.* | 43 y. | 3 m. | u. | | p. |
| 254 " | " | " | *R.* | 29 y. | 1 y. | u. | | ip. |
| 255 " | " | " | J. D. B. | 56 y. | 4 m. | u. | | ip. |
| 256 Ulna, | 3 points | simp.&com. | J. L. | 25 y. | 2 y. | u. | 1 in. | ip. |
| 257 " | up. ⅓ | simple | J. C. | 18 y. | 1 y. | u. | | p. |

230. Had been broken before at same point and left deformed; remained in same condition after second fracture; false anchylosis of elbow joint.

232. Could not flex fore-arm upon humerus for six months; now motion perfect, but arm pains when used.

233. Also fracture of lower end of humerus at same time. Cannot extend, or flex fore-arm, more than to right angle; flexion of fingers imperfect. Arm pains when she attempts to use it.

235. There was false anchylosis of elbow joint for a long time; now motion free.

236. Outer condyle is separated half an inch; deformity evident; cannot straighten arm perfectly.

237. Much deformed; hand forcibly drawn toward radial side.

239. Upper end of lower fragment displaced forward; supination lost; flexion very imperfect.

242. Did not employ a surgeon for the first five weeks.

243. Pronation and supination imperfect.

244. Somewhat bent at seat of fracture.

246. This was a bend of radius and fracture of ulna. No. 261.

250. Radius was bent forward at seat of fracture when splints were removed, but, as is often the case with young persons, time has made it nearly perfect.

252. Deformity great; hand bent backward and very weak.

254. Slightly bent; motions of hand impaired.

255. Slight displacement of ulna; hand stiff, swollen and partly paralyzed.

256. Remains bent.

| Name of Bones. | Point of Fracture. | Character of Frac. | Name of Patient. | Age then. | Time since it occurred. | Unit'd or not united | Amt. of short'nlng. | Perfect or imp'rfect |
|---|---|---|---|---|---|---|---|---|
| 258 Ulna, | up. $\frac{1}{3}$ | simple | U. L. | 40 y. | 1 y. | u. | | ip. |
| 259 " | " | " | *R. S.* | 46 y. | 9 w. | u. | | ip. |
| 260 " | middle | " | S. G. | 30 y. | 10 y. | u. | | p. |
| 261 " | " | " | P D. | $1\frac{2}{3}$ y. | 4 m. | u. | | p. |
| 262 " | " | " | M. O. B. | 3 y. | 6 w. | u. | | p. |
| 263 " | up. $\frac{1}{8}$ | " | *E. C.* | $3\frac{2}{3}$ y. | 2 m. | u. | | p. |
| 264 " | low $\frac{1}{3}$ | " | O. H. P. | 26 y. | 11 w | u. | | ip. |
| 265 " | low $\frac{1}{8}$ | " | P. W. | 39 y. | 1 y. | u. | | ip. |
| 266 " | olecranon P | " | J. C. | 18 y. | 9 y. | u. | | ip. |
| 267 " | " | " | P. C. | 14 y. | 69 y. | u. | | ip. |
| 268 " | " | " | S. D. | 14 y. | 5 w. | u. | | p. |
| 269 " | " | " | C. A. | 15 y. | 6 m. | u. | | ip. |
| 270 Rad. & ulna | low 1-6 | compound | *A. F.* | 11 y. | 1 y. | u. | | p. |
| 271 " | up $\frac{1}{3}$ | simple | W. C. | 4 y. | 20 y. | u. | | p. |
| 272 " | middle | " | D. M. | 14 y. | 4 y. | u. | $\frac{1}{4}$ in. | ip. |
| 273 " | " | " | I. S. | 9 y. | 17 y. | u. | $\frac{3}{8}$ in. | ip. |
| 274 " | " | " | J. M. C. | 63 y. | 3 y. | u. | | p. |
| 275 " | " | " | T. B. S. | 11 y. | 11 y. | u. | | p. |
| 276 " | " | " | J. W. | 10 y. | 40 y. | u. | | p. |
| 277 " | " | " | W. P. | 12 y. | 14 y. | u. | | p. |
| 278 " | " | " | J. B. | 15 y. | 25 y. | u. | | p. |
| 279 " | " | " | I. H. L. | 37 y. | 40 y. | u. | | p. |
| 280 " | " | " | G. B. | 11 y. | 5 w. | u. | | p. |
| 281 " | " | " | *M. C.* | 9 y. | 10 y. | u. | | p. |
| 282 " | low $\frac{1}{3}$ | " | J. J. | 10 y. | 42 y. | u. | | p. |
| 283 " | " | " | M. B. | 23 y. | 2 m. | u. | | p. |
| 284 " | " | " | M. | 3 y. | 8 w. | u. | | p. |
| 285 " | " | " | H. A. C. | 12 y. | 6 y. | u. | | p. |
| 286 " | " | " | P. W. T. | 32 y. | 4 m. | u. | | p. |
| 287 " | " | " | *E. McL.* | 1 y. | 3 y. | u. | | p. |
| 288 " | low $\frac{1}{8}$ | " | P. H. | 30 y. | 10 y. | u. | | ip. |

258. Head of radius dislocated forward, unreduced ; motion nearly perfect.

259. Slight bend at seat of fracture; lower fragment overlaps upper; wrist anchylosed.

261. See No. 246.

264. Anterior luxation of head of radius, unreduced, ulna bent; motions perfect; head of radius does not strike humerus.

266. United by ligament; arm as strong as before.

267. Cannot straighten arm perfectly ; pronation and supination imperfect.

268. Union appears to be perfect ; four weeks before had a fracture of radius of same arm, which was treated by an empiric.

269. Radius dislocated forward ; cannot straighten arm.

271. See No. 222 This was a bend of both bones and not a complete fracture.

272. Arm not as strong as before and at times painful.

274. Same patient as No. 217.

277. Flexion and supination imperfect.

279. Pronation and supination imperfect.

283. Same patient as No. 214. Both fractures caused by the crank of a hand car when under full speed ; slight bend at seat of fracture.

284. This was a bend of both bones.

285. Radius bent forward at seat of fracture.

286. Four months since, broke radius and ulna, and three months after refractured them at same point. Now but slight bend of ulna, at seat of fracture.

288. Use of arm perfect ; did not know it was shortened.

| Name of Bones. | Point of Fracture. | Character of Frac. | Name of Patient. | Age then. | Time since it occurred. | Unit'd or not united | Amt. of short'ning. | Perfect or imp'rfect |
|---|---|---|---|---|---|---|---|---|
| 289 Rad. & ulna | low $\frac{1}{8}$ | simple | M. C. | 40 y. | 5 w. | u. | | p. |
| 290 " | " | " | P. McE. | 8 y. | | | | p. |
| 291 Carpal | | comp. | S. L. | 18 y. | 1 y. | u. | | ip. |
| 292 Metacarpal of thumb | up $\frac{1}{3}$ | simple | M. H. | 26 y. | 2 m. | u. | | ip. |
| 293 do. of finger | low $\frac{1}{3}$ | comp. | H. H. | 21 y. | 1 y. | u. | | p. |
| 294 " " | low $\frac{1}{4}$ | simple | W. P. | 27 y. | 14 y. | u. | | ip. |
| 295 2d and 3d phalanges | | comp. | J. T. | 7 y. | 1 y. | u. | | ip. |
| 296 Ensiform cartilage | base | simple | H. B. R. | 28 y. | 12 y. | u. | | ip. |
| 297 7th cerv. vertebra | spin. process | " | R. L. | 50 y. | 9 w. | u. | | ip. |
| 9 8 Lumbar " | | " | —— | 45 y. | 36 y. | u. | | |
| 299 Lumbar | | " | B. | 25 y. | 15 y. | u. | | |
| 300 Os innominatum | | " | P. C. | 40 y. | | u. | | ip. |
| 301 Acetabulum | | " | M. S. | 40 y. | | | $1\frac{1}{2}$ in | ip. |
| 302 Femur | middle | comp. com. | J. A. B. | 14 y. | 17 y. | u. | 1 in. | ip. |
| 303 " | " | " | P. H. | 23 y. | 38 y. | u. | $1\frac{1}{4}$ in | ip. |
| 304 " | low $\frac{1}{8}$ | " | D. W. | 20 y. | 5 y. | u. | 7 in. | ip. |
| 305 " | middle | compound | J. S. | 18 y. | 10 w | u. | 1 in. | ip. |
| 306 " | neck of f. | simple | J. B. | 52 y. | 3 m. | u. | $1\frac{1}{2}$ in | ip. |
| 307 " | " | " | T. K. | 77 y. | 1 y. | u. | 3 in. | ip. |
| 308 " | " | " | J. S. | 39 y. | 6 y. | u. | 2 in. | ip. |
| 309 " | " | " | L. F. T. | 42 y. | 6 w. | u. | $\frac{1}{2}$ in. | ip. |
| 310 " | " | " | W. | 66 y. | 5 w. | u. | | ip. |
| 311 " | troc. mag. | " | B. R. | 25 y. | 2 m. | u. | | ip. |

291. While holding right hand over muzzle of a gun, it was discharged, and a charge of shot passed through the *semilunare, cuneiforme and unciforme,* removing part of these bones—can move all fingers except little finger.

292. Upper end of lower fragment is displaced slightly inward.

294. Fragments somewhat displaced.

295. Anchylosis—slight bend at seat of fracture; still very tender.

302. Did not use limb for one year. After eighteen months a piece of bone was removed. No halt.

304. Right femur was crushed by a heavy weight. Several pieces came out. Did not know limb was shortened more than three inches; by means of one inch added to the heel of right shoe, he walks with hardly any perceptible halt. When standing the ala of right side of pelvis is four inches lower than left and becomes almost six inches when he walks. This inclination of the pelvis is accomplished by a lateral curvature of the lumbar vertabræ.

| Name of Bones. | Point of Fracture. | Character of Frac. | Name of Patient. | Age then. | Time since it occurred. | Unit'd or not united | Amt. of short'ning | Perfect or imp'rfect |
|---|---|---|---|---|---|---|---|---|
| 312 Femur | up $\frac{1}{3}$ | simple | M. C. | 9 y. | 31 y. | u. | $1\frac{1}{2}$ in | ip. |
| 313 " | " | " | A. G. | 35 y. | 6 w. | n. | $\frac{3}{4}$ in. | ip. |
| 314 " | " | " | J. C. | 49 y. | 7 w. | u. | $\frac{1}{2}$ in. | ip. |
| 315 " | " | " | I. H. F. | 30 y. | 16 d. | u. | $3\frac{7}{8}$ in | ip. |
| 316 " | " | " | J. R | 40 y. | 1 m. | u. | | ip. |
| 317 " | " | " | E. R. | 42 y. | 4 y. | u. | $\frac{3}{4}$ in. | ip. |
| 318 " | " | " | J. M. | 1 y. | 1 y. | u. | | p. |
| 319 " | middle | " | J. G. | 8 y. | 22 y. | u. | $\frac{1}{2}$ in. | ip. |
| 320 " | " | " | J. G. | 14 y. | 16 y. | u. | $\frac{1}{2}$ in. | ip. |
| 321 " | " | " | M. H. | 15 y. | 19 y. | u. | $\frac{1}{2}$ in. | ip. |
| 322 " | " | " | H. C. | 16 y. | 25 y. | u. | $\frac{3}{4}$ in. | ip. |
| 323 " | " | " | D. K. | 15 y. | 8 y. | u. | | p. |
| 324 " | " | " | A. A. | 22 y. | 9 w. | u. | $\frac{1}{2}$ in. | ip. |
| 325 " | " | " | A. S. | 13 y. | 19 y. | u. | $1\frac{1}{4}$ in | ip. |
| 326 " | " | " | S. B. | 3 y. | 63 y. | u. | | p. |
| 327 " | " | " | J. G. | 13 y. | 6 w. | u. | | p. |
| 328 " | " | " | G. W. H. | 43 y. | 6 w. | u. | $\frac{1}{2}$ in. | ip. |
| 329 " | " | " | M. | 3 y. | 5 w. | u. | | p. |
| 330 " | " | " | P. J. | 15 y. | 30 y. | u. | | p. |
| 331 " | " | " | G. S. L. | 2[illegible] | 1 y. | u. | $\frac{1}{2}$ in. | ip. |
| 332 " | " | " | E. S. | 8 y. | 5 y. | u. | $\frac{1}{2}$ in. | ip. |
| 333 " | " | " | R. | 9 y. | 3 m. | u. | | p. |
| 334 " | " | " | M. | 18 y. | 8 w. | u. | | p. |
| 335 " | " | " | J. McE. | 22 y. | 1 y. | u. | 3 in. | ip. |
| 336 " | " | " | J. L. | 35 y. | 1 y. | u. | $\frac{3}{4}$ in. | ip. |
| 337 " | " | " | J. L. | 35 y. | 3 m. | u. | $\frac{3}{4}$ in. | ip. |

312. The overlaping of fragments at seat of fracture is very perceptible; has but slight halt.

313. Had been fractured before at same point; was shortened then, but more now; straight splint was used and adhesive plaster extension.

315. Fourteen years since, broke left femur by a fall, while walking; an empiric treated it nine weeks, with short splints, without obtaining union, patient then went to another empiric who treated it with pasteboard and leather splints, and in five weeks he was about. About one year after he refractured same bone; it was treated in same manner by the empiric; patient was up in fourteen days. Eight years since, he hurt same limb; empiric said it was broke. Sixteen days since, he broke it a fourth time by jumping from a wagon. A surgeon was then called for the first time. Now united, but much bent at seat of fracture.

318. Bent forward at seat of fracture; toes turned in a little.

319. 320. Same patient; both fractures near same point; straight splint was used.

322. No halt can be perceived in his walk.

323. Straight splint was used.

324 Straight splint and adhesive plaster extension.

325. No deformity; no halt.

327. Straight splint and starch bandage was used.

328. Straight splint, and after three weeks the double inclined plane.

329. Dressed with roller and laid in a pillow.

332. Double inclined plane was used.

333. 334. Straight splint used, also in No. 331.

335. Union was delayed about eight weeks; four months from the first fracture patient refractured it, by turning in bed, to which he was confined by a nonunited fracture of tibia and fibula. See No. 388. Union took place with great deformity after each fracture.

336. 337. Same patient. Straight splint was used each time.

| Name of Bones. | Point of Fracture. | Character of Frac. | Name of Patient. | Age then. | Time since it occurred. | Unit'd or not united | Amt. of short'ning. | Perfect or imp'rfect |
|---|---|---|---|---|---|---|---|---|
| 338 Femur | middle | simple | D. C. | 29 y. | 1 y. | u. | $\frac{3}{4}$ in. | ip. |
| 339 " | " | " | J. T. | 14 y. | 12 w | u. | $\frac{3}{4}$ in. | ip. |
| 340 " | " | " | *S. S. S.* | 27 y. | 6 w. | u. | | ip. |
| 341 " | low $\frac{1}{3}$ | " | A. M. | 24 y. | 30 y. | u. | $1\frac{3}{4}$ in | ip. |
| 342 " | " | " | J. B. | 8 y. | 4 w. | u. | | p. |
| 343 " | " | " | P. H. | 29 y. | 4 m. | u. | | p. |
| 344 " | " | " | E. S. | 42 y. | 5 y. | u. | 1 in. | ip. |
| 345 " | " | " | H. H. | 42 y. | 7 w. | u. | $\frac{1}{2}$ in. | ip. |
| 346 Patella | trans. | " | E. L. | 20 y. | 7 w. | u. | | p. |
| 347 " | " | " | J. McC. | 33 y. | 2 y. | u. by lig. | | ip. |
| 348 " | " | " | W. | 40 y. | 9 w. | " | | ip. |
| 349 " | " | " | C. A. | 31 y. | 4 m. | u. | | ip. |
| 350 " | " | " | J. D. | 36 y. | 3 m. | u. | | ip. |
| | | | | | | u. | | |
| 351 Tibia | middle | comp. | R. P. | 53 y. | 4 y. | u. | | p. |
| 352 " | " | " | S. C. | 28 y. | 7 m. | u. | $\frac{1}{4}$ in. | ip. |
| 353 " | low $\frac{1}{8}$ | " | P. M. | 28 y. | 4 m. | u. | | ip. |
| 354 " | " | " | J. T. | 17 y. | 6 y. | u. | | ip. |
| 355 " | " | " | J. McD. | 38 y. | 3 y. | u. | | p. |
| 356 " | up $\frac{1}{8}$ | simple | J. E. | 42 y. | 10 y. | u. | | p. |
| 357 " | up 1-6 | " | R. P. | 55 y. | 7 y. | u. | $\frac{1}{4}$ in. | ip. |
| 358 " | middle | " | R. J. | 46 y. | 10 m | u. | | p. |
| 359 " | " | " | G. P. | 11 y. | 6 w. | u. | | p. |
| 360 " | " | " | G. E. | 17 y. | 11 y. | u. | | p. |
| 361 " | " | " | M. H. | 30 y. | 4 y. | u. | | p. |
| 362 " | " | " | H. W. | 17 y. | 4 y. | u. | | p. |
| 363 " | " | " | J. T. | 11 y. | 16 y. | u. | | p. |
| 364 " | " | " | G. G. | 18 y. | 7 y. | u. | | p. |
| 365 " | " | " | *L.* | 50 y. | 2 m. | u. | | p. |
| 366 " | low $\frac{1}{8}$ | " | M. C. | 19 y. | 2 m. | u. | | p. |
| 367 " | mall. int. | " | W. G. | 35 y. | 6 w. | u. | | p. |

338. Leg pains him when used ; not as strong as before.
339. Union did not take place in eleven weeks. Straight splint used.
340. Limb was shortened. Straight splint used.
341. Double inclined plane used.
342. 344. 345. Straight sp'int used.
347. Had splint on twenty-five days. Union by ligament $\frac{1}{2}$ inch in length.
348. Had splint on twenty-eight days ; five weeks after first fracture refractured it while walking.
349. Sixteen weeks since, fractured patella, and eight weeks after refractured it at same point. Union by ligament $\frac{1}{4}$ inch in length as in No 348 and No. 350.
352. Seven months after the accident, limb was quite lame, but improved after the removal of a small fragment of bone which was found to be loose.
353. Fracture extended into joint ; astragalus and lower end of tibia became necrosed; ankle but little stiff.
356. A little bent at seat of fracture.
357. Same patient as No. 351, and also same leg. Head of fibula displaced ; tibia bent at seat of fracture ; limb pains when he walks; also see No. 374.
358. Slight bend at seat of fracture ; pains him when used.
366. This patient at same time fractured the tibia and fibula of other leg, see No. 399. The starch bandage was here used as in case of No. 359 and No. 365.

| Name of Bones. | Point of Fracture. | Character of Frac. | Name of Patient. | Age then. | Time since it occurred. | Unit'd or not united | Amt, of short'ning. | Perfect or imp'rfect |
|---|---|---|---|---|---|---|---|---|
| 368 Fibula | middle | comp. com. | T. M. | | | n. u. | | ip. |
| 369 " | " | comp. | R. C. | 20 y. | 5 y. | u. | | p. |
| 370 " | " | " | H. W. | 28 y. | 14 y. | u. | | p. |
| 371 " | up ⅛ | simple | —— | 60 y. | 3 w. | u. | | p. |
| 372 " | low ⅓ | " | C. B. | 34 y. | 14 y. | u. | | p. |
| 373 " | " | " | I. S. | 34 y. | 28 y. | u. | | p. |
| 374 " | low ⅛ | " | R. P. | 57 y. | 4 w. | u. | | p. |
| 375 " | " | " | J. R. | 32 y. | 3 m. | u. | | ip. |
| 376 " | " | " | H. B. | | | u. | | ip. |
| 377 " | " | " | J. S. | 10 y. | 4 y. | u. | | ip. |
| 378 " | " | " | J. S. | 32 y. | 20 y. | u. | | ip. |
| 379 " | ext. mall. | " | A. G. | 21 y. | 8 m. | u. | | ip. |
| 380 Tib. & Fib. | middle | comp. com, | J. O. | 48 y. | 3 m. | u. | ½ in. | ip. |
| 381 " | low ⅓ | " | H. K. | 14 y. | 7 m. | u. | 2 in. | ip. |
| 382 " | low 1-6 | " | C. S. | 50 y. | 6 m. | u. | | ip. |
| 383 " | low ⅛ | " | J. B. F. | 39 y. | 6 y. | u. | 1 in. | ip. |
| 384 " | up ⅓ | comp. | I. R. | 34 y. | 8 y. | u. | ¾ in. | ip. |
| 385 " | middle | " | T. C. | 4 y. | 23 y. | u. | ½ in. | ip. |
| 386 " | " | " | J. W. | 11 y. | 1 y. | u. | | p. |
| 387 " | " | " | W. K. | 35 y. | 4 m. | u. | | p. |
| 388 " | " | " | J. McE. | 22 y. | 1 y. | u. | 1½ in | ip. |
| 389 " | low ⅓ | " | A. R. | 30 y. | 2 y. | u. | ½ in. | ip. |
| 390 " | " | " | M. F. | 24 y. | 3 m. | u. | | ip. |
| 391 " | " | " | G. W. B. | 33 y. | 1 y. | u. | ½ in. | ip. |
| 392 " | " | " | W. H. | 32 y. | 22 y. | u. | | p. |

371. The tendency to displacement was so little, that no splint was used ; the horizontal posture was sufficient to keep fragments in place—caused by direct blow—no displacement of tibia.

374. See No. 351 ; 357 same patient.

375. Tibia was dislocated inward ; still remains slightly displaced. Fibula bent at seat of fracture. Ankle swollen.

376. Tibia dislocated inward, unreduced.

377. Foot turned slightly out ; some enlargement above malleolus externus.

378. There is some deformity ; ankle at times becomes swollen and painful.

379. Foot turns in ; lower fragment displaced downward.

380. Starch bandage and the double inclined place was used. Union was not complete on the 25th day, but patient was allowed to get up on 39th day. Dr. H. saw him again two years after and found ankle and knee stiff.

382. Bones united in four weeks. Had ulcer on heel.

384. Both bones much bent at seat of fracture.

385. Has remained fistulous, fragments of bone have frequently been removed from the wound.

386. Starch bandage used.

388. On the first day of work after an attack of bilious fever, a bank of earth fractured his left tibia and fibula, and also his right femur, see No. 335. Soon ulcers formed on both heels ; about thirteen weeks after accident erysipelas appeared on the left leg. Union took place in eleven months, with deformity ; the lower end of upper fragments override the upper end of lower fragments.

389. Bones project at seat of fracture. Six months since a slight injury at that point produced an ulcer, which is still open.

390. Bones much bent, lower end of upper fragment projects anteriorly. Heel bent backward. Skin over seat of fracture red and tender, appears as if it would ulcerate.

391. Ankle stiff and swollen. Patient did not know limb was shortened.

| Name of Bones. | Point of Fracture. | Character of Frac. | Name of Patient. | Age then. | Time since it occurred. | Unit'd or not united | Amt. of short'ning. | Perfect or imp'rfect |
|---|---|---|---|---|---|---|---|---|
| 393 Tib. & Fib. | low 1/3 | comp. | I. O. B. | 26 y. | 5 m. | u. | 3/4 in. | ip. |
| 394 " | " | " | P. B. | 25 y. | 6 m. | u. | 1/2 in. | ip. |
| 395 " | " | " | M. S. | 40 y. | —— | n. u. | | ip. |
| 396 " | " & up 1-6 | " | B. G. McK. | 39 y. | 17 y. | u. | 1 1/4 in | ip. |
| 397 " | low 1/8 | " | M. F. | 31 y. | 2 y. | n. u. | | ip. |
| 398 " | up 1/8 | simple | —— | 25 y. | 8 w. | u. | | p. |
| 399 " | mid.& low 1/8 | simp.& com. | M. C. | 19 y. | 2 m. | u. | | p. |
| 400 " | up 2/3 | simple | E. T. | 21 y. | 5 y. | u. | 5/8 in. | ip. |
| 401 " | " | " | T. L. | —— | —— | u. | 1/2 in. | ip. |
| 402 " | middle | " | W. B. | 38 y. | 8 m. | u. | 1/2 in. | ip. |
| 403 " | " | " | E. V. | 10 y. | 15 w | u. | | p. |
| 404 " | " | " | W. McC. | 19 y. | 3 m. | u. | | p. |
| 405 " | " | " | W. R. | 38 y. | 5 w. | u. | | p. |
| 406 " | " | " | J. M. | 16 y. | 8 w. | u. | | p. |
| 407 " | low 1/3, up 1/8 | " | R. S. | 46 y. | 4 m. | n. u. | 1/2 in. | ip. |
| 408 " | low 1/3 | " | I. H. L. | 52 y. | 25 y. | u. | 1/2 in. | ip. |
| 409 " | " | " | J. H. L. | 15 y. | 5 w. | u. | | p. |
| 410 " | " | " | E. McD. | 35 y. | 6 w. | u. | 3/4 in. | ip. |
| 411 " | " | " | A. T. | 49 y. | 2 y. | u. | 3/4 in. | ip. |
| 412 " | " | " | T. F. | 38 y. | 2 y. | u. | 1 1/4 in | ip. |
| 413 " | " | " | T. S. | 40 y. | 8 w. | u. | 1/2 in. | ip. |
| 414 " | " | " | T. H. | 3 y. | 6 w. | u. | | p. |
| 415 " | " | " | I. C. C. | 14 y. | 8 y. | u. | | p. |
| 416 " | " | " | J. K. | 16 y. | 8 m. | u. | | p. |
| 417 " | " | " | J. J. | 22 y. | 6 m. | u. | | p. |
| 418 " | " | " | A. A. H. | 32 y. | 1 y. | u. | 1/2 in. | ip. |
| 419 " | " | " | J. H. | 25 y. | 2 y. | u. | 1/4 in. | ip. |
| 420 " | " | " | *I. P. W.* | —— | 4 m. | u. | | p. |
| 421 " | low 1-6 | " | E. B. | 42 y. | 8 y. | u. | 1/4 in. | ip. |
| 422 " | low 1/3 | " | E. B. | 50 y. | 3 m. | u. | 1/4 in. | ip. |
| 423 " | low 1-6 | " | I. T. | 44 y. | 24 y. | n. | | p. |

393. A fragment of bone was removed.

394. Health bad before accident. Much bent at seat of fracture, has an ulcer over seat of fracture.

395. The attempt was made for fifteen days to save limb, then amputation was performed.

396. Lower end of upper fragment of tibia projects over lower fragment. Patient did not rest any weight upon his limb until after four months, and it was not until after the fifth month that he attempted to do without his crutches. Pains him at seat of fracture; motion of ankle not quite as free as the other.

397. Amputated five weeks after fracture.

399. See No. 367: starch bandage used in both fractures.

402. Upper end of lower fragment is anterior to lower end of upper; foot weak: uses crutch. Here as in No. 266 and No. 267, the starch bandage was used.

406. There was no displacement of the bones.

407. Lower end of upper fragment is anterior to upper end of lower fragment; union not yet complete; used extension twenty-five days, then starch bandage. Had ulcer on heel.

408. Bent at seat of fracture; ankle stiff, enlarged; pains more and more each year.

410. There is an anterior and lateral displacement of the lower end of upper fragment.

411. It remains much swollen.

413. 416. Slightly bent at seat of fracture.

421. 422. These are one person.

| Name of Bones. | Point of Fracture. | Character of Frac. | Name of Patient. | Age then. | Time since it occurred. | Unit'd or not united | Amt. of short'ning. | Perfect or imp'rfect' |
|---|---|---|---|---|---|---|---|---|
| 424 Tib. & Fib. | low $\frac{1}{8}$ | simple | A. V. | 37 y. | 25 y. | u. | $\frac{1}{2}$ in. | ip. |
| 425 " | " | " . | H. M. | 43 y. | 10 w | u. | $\frac{1}{2}$ in. | ip. |
| 426 " | " | " | E. H. | 27 y. | 6 w. | u. | | ip. |
| 427 " | " | " | *M. H.* | 54 y. | 4 w. | u. | $\frac{1}{4}$ in. | ip. |
| 428 " | low 1-6 | " | J. H. | $6\frac{2}{3}$ y. | 4 m. | u. | | p. |
| 429 " | low 1-6 and int. mall. | " | I. B. | 33 y. | 30 y. | u. | | p. |
| 430 " | up $\frac{1}{8}$ | " | F. C. T. | 35 y. | 8 w. | n. u. | $\frac{3}{4}$ in. | ip. |

424. Foot turned in very much.

429. Ankle anchylosed. Pains him more now than it did years ago; limb emaciated.

430. This patient, never a stout, robust man, is now pale, skin cold and moist; under excitement his pulse varies in a short time, from 100 to 140. Leg enormously swollen and œdematous; no union; upper end of lower fragment is in front of lower end of upper fragment. Has been treated with the lateral splints.

The following *thirty-one* cases are drawn also from Dr. H.'s notes, being cases in which death or amputation followed the fracture, and on that account were not admitted to the other tables:

| Name of Bone. | Point of Fracture. | Character of Frac. | Name of Patient. | Age then. | Time of Amputa'n. | Point of Amputa'n. | Result. | Time of Death. |
|---|---|---|---|---|---|---|---|---|
| 431 Inf. Maxilla, | | comp. com. | J. G. | 31 y. | | up $\frac{1}{3}$ | d. | 12th d. |
| 432 Humerus, | up $\frac{1}{3}$ | " | K. | 30 y. | 17 d. | " | | |
| 433 " | middle | " | E. H. | 21 y. | 19 h. | " | r. | |
| 434 " | | " | E H. | 21 y. | 18 h. | " | r. | |
| 435 " | " | " | W. L. | 28 y. | 4 h. | shoulder | r. | |
| 436 " | " | simple | J. C. | 35 y. | | " joint | r. | |
| 437 Radius & Ulna, | low $\frac{1}{3}$ | comp. com. | I. | 27 y. | 6 h. | middle | r. | |
| 438 " | middle | " | F. P. | 19 y. | 14 h. | humerus | d. | 15 h. |
| 439 Carpus, etc., | | " | Hollander, | 11 y. | soon | low $\frac{1}{2}$ of rad. and ulna | d. | 36 h. |
| 440 " | | " | H. C. | 20 y. | " | rad. & carp. artic. | r. | |
| 141 " | | " | H. C. | 22 y. | 12 h. | wrist joint | r. | |
| 442 Phalanx, | 2d | " | Boy, | 12 y. | 18 h. | 2d artic. | r. | |
| 443 " | 2d | " | " | 12 y. | " | mid. 2d phal. | r. | |
| 444 " | 3d | " | B. | 20 y. | soon | last artic | r. | |
| 445 " | 1st | " | P. H. | 28 y. | " | m.carp artic. | r. | |
| 446 " | 1st | " | " | 28 y. | " | " | r. | |
| 447 " | 2d | " | " | 28 y. | " | 1st phalnx. | r. | |
| 448 " | 3d | " | B. | 40 y. | 6 h. | 2d artic. | r. | |
| 449 " | 2d and 3d | " | S. C. | 25 y. | 5 w. | 1st artic. | r. | |
| 450 " | " | " | L. R. | 30 y. | 12 h. | " | r. | |
| 451 " | " | " | " | 30 y. | " | " | r. | |
| 452 " | " | " | " | 30 y. | " | " | r. | |
| 453 " | 1st, 2d & 3d | " | J. W. | 45 y. | 4 w. | metacarpus | r. | |
| 454 Femur, | neck | comp. | J. O'K. | 40 y. | | | d. | 6 h. |
| 455 " | middle | comp com. | H. B. | 21 y. | | | d. | 34 b. |
| 456 " | " | simple | " | 21 y. | | | d. | 34 h. |
| 457 " | " | commin. | J. N. | 30 y. | 5 h. | up $\frac{1}{3}$ | d | 8 h. |
| 458 " | — | comp. com. | A, M. | 30 y. | 5 w. | middle | r. | |
| 459 Tibia & Fibula, | " | " | J. S. | 30 y. | " | | | |
| 460 " | " | " | I. B. | 3 y. | soon | up $\frac{1}{3}$ | d. | 9 d. |
| 461 " | up $\frac{1}{3}$ | " | D. R. | 40 y. | 7 h. | above knee | d. | 3 h. |

### SUMMARY.

| Of the Cases Reported by Dr. Hamilton. — Name of Bones. | No. of Fractures. | No. of Perfect. | No. of Imperfect. | Points of Fracture. — Upper or outer $\frac{1}{3}$. | Middle. | Lower $\frac{1}{3}$. | Character of Fracture. — Comp. & Commin. | Comp. | Simple | Relative No. at all Ages. — To 15 years. No | P. | 15 to 30 years. No | P. | 30 to 45 years. No | P | 45 to 60 years. No | P. | 60 to 82 years. No | P. |
|---|---|---|---|---|---|---|---|---|---|---|---|---|---|---|---|---|---|---|---|
| Ossa Nasi, | 3 | .. | 3 | .. | .. | .. | .. | 1 | 2 | 2 | .. | 1 | .. | .. | .. | .. | .. | .. | .. |
| Vomer, | 1 | .. | 1 | .. | .. | .. | .. | .. | 1 | 1 | .. | .. | .. | .. | .. | .. | .. | .. | .. |
| Inf. Maxilla, | 5 | 3 | 2 | * | .. | .. | 5 | .. | .. | 1 | 1 | 2 | 2 | 1 | .. | 1 | .. | .. | .. |
| Clavicle, | 18 | 6 | 12 | 15 | 3 | .. | .. | 4 | 14 | 3 | 2 | 7 | 2 | 7 | 2 | 1 | .. | .. | .. |
| Scapula, | 1 | .. | 1 | † | .. | .. | .. | .. | 1 | .. | .. | 1 | .. | .. | .. | .. | .. | .. | .. |
| Humerus, | 14 | 5 | 9 | 1 | 2 | 11 | 2 | .. | 12 | 8 | 4 | 2 | .. | 3 | .. | 1 | 1 | .. | .. |
| Radius, | 8 | 5 | 3 | 1 | 1 | 6 | .. | .. | 8 | 4 | 3 | 2 | 2 | 2 | .. | .. | .. | .. | .. |
| Ulna, | 9 | 8 | 1 | 2 | 3 | 4 | .. | 2 | 7 | 4 | 4 | 3 | 3 | 2 | 1 | .. | .. | .. | .. |
| Radius & Ulna, | 13 | 7 | 6 | 1 | 1 | 10 | .. | 1 | 11 | 8 | 5 | 3 | 2 | 2 | .. | .. | .. | .. | .. |
| Femur, | 29 | 4 | 25 | 10 | 13 | 1 | .. | .. | 27 | 7 | 2 | 6 | 1 | 9 | 1 | 3 | .. | 4 | .. |
| Patella, | 2 | .. | 2 | .. | .. | .. | .. | .. | 2 | 1 | .. | 1 | .. | .. | .. | | .. | .. | .. |
| Tibia, | 2 | 1 | 1 | 1 | .. | 1 | .. | 1 | 1 | .. | .. | 1 | .. | .. | .. | 1 | 1 | .. | .. |
| Fibula, | 4 | 2 | 2 | .. | .. | 4 | .. | 2 | 2 | 1 | .. | .. | .. | 3 | 2 | .. | .. | .. | .. |
| Tibia and Fibula, | 22 | 5 | 17 | 2 | 9 | 10 | 9 | 5 | 7 | 6 | 2 | 5 | 1 | 10 | 2 | 1 | .. | .. | .. |
| Amount, | 131 | 46 | 85 | 33 | 32 | 47 | 16 | 16 | 95 | 46 | 23 | 34 | 13 | 39 | 8 | 8 | 2 | 4 | .. |
| *Of the Supplement.* | | | | | | | | | | | | | | | | | | | |
| Ossa Nasi, | 6 | 1 | 5 | .. | .. | .. | .. | 2 | 4 | 3 | 1 | 2 | .. | .. | .. | .. | .. | .. | .. |
| Septum Nasi, | 3 | .. | 3 | .. | .. | .. | .. | .. | 3 | 2 | .. | 1 | .. | .. | .. | .. | .. | .. | .. |
| Sup. Maxilla, | 1 | 1 | .. | .. | .. | .. | .. | .. | 1 | .. | .. | .. | .. | 1 | 1 | .. | .. | .. | .. |
| Inf. Maxilla, | 8 | 4 | 4 | .. | .. | .. | 1 | 3 | 1 | 1 | 1 | 5 | 2 | 2 | 1 | .. | .. | .. | .. |
| Clavicle, | 23 | 9 | 14 | 17 | 6 | .. | .. | .. | 23 | 7 | 6 | 2 | 1 | 7 | .. | 6 | 2 | .. | .. |
| Scapula, | 2 | 1 | 1 | ‡ | ‖ | .. | .. | .. | 2 | .. | .. | 1 | .. | .. | .. | .. | .. | 1 | 1 |
| Humerus, | 24 | 12 | 12 | 4 | 5 | 15 | .. | 1 | 24 | 10 | 4 | 3 | 3 | 7 | 4 | 2 | 1 | 2 | .. |
| Radius, | 19 | 12 | 7 | 2 | 3 | 14 | .. | 2 | 17 | 8 | 6 | 5 | 3 | 4 | 2 | 2 | .. | .. | .. |
| Ulna, | 14 | 6 | 8 | § | 3 | 2 | .. | .. | 13 | 6 | 4 | 5 | 2 | 2 | .. | 1 | .. | .. | .. |
| Radius and Ulna, | 21 | 18 | 3 | 1 | 10 | 10 | .. | 1 | 20 | 15 | 13 | 2 | 1 | 3 | 3 | .. | .. | 1 | 1 |
| Femur, | 44 | 10 | 34 | 13 | 25 | 6 | 3 | 1 | 40 | 16 | 8 | 4 | 2 | 10 | .. | 2 | .. | 2 | .. |
| Patella | 5 | 1 | 4 | .. | .. | .. | .. | .. | 5 | .. | .. | 1 | 1 | 4 | .. | .. | .. | .. | .. |
| Tibia, | 17 | 13 | 4 | 2 | 10 | 5 | .. | 5 | 12 | 2 | 2 | 8 | 5 | 3 | 3 | 4 | 3 | .. | .. |
| Fibula, | 12 | 6 | 6 | 1 | 3 | 8 | 1 | 2 | 9 | 1 | .. | 4 | 2 | 3 | 2 | 2 | 2 | .. | .. |
| Tibia and Fibula, | 51 | 18 | 33 | 5 | 11 | 35 | 4 | 14 | 33 | 5 | 3 | 10 | 4 | 12 | 3 | .. | .. | .. | .. |
| Amount, | 250 | 112 | 138 | 45 | 76 | 92 | 8 | 31 | 207 | 76 | 48 | 53 | 26 | 58 | 19 | 19 | 8 | 6 | 2 |

* 5 Shaft. † Acro. p. ‡ Corocoid p. 1. § Olec. 4. ‖ Below spine.

SUMMARY OF TABLES OF ALL THE CASES FROM THE NOTES OF DR. HAMILTON.

| Name of Bones. | No. of Fractures. | No. of Perfect. | No. of Imperfect. | Points of Fracture. Upper or outer ⅓. | Points of Fracture. Middle. | Points of Fracture. Lower ⅓. | Character of Fracture. Comp & Commin | Character of Fracture. Comp. | Character of Fracture. Simple. | To 15 years. No. | To 15 years. P. | 15 to 30 years. No | 15 to 30 years. P. | 30 to 45 years. No | 30 to 45 years. P. | 45 to 60 years. No | 45 to 60 years. P. | 60 to 82 years. No | 60 to 82 years. P. |
|---|---|---|---|---|---|---|---|---|---|---|---|---|---|---|---|---|---|---|---|
| Ossa Nasi, | 9 | 1 | 8 | .. | .. | .. | .. | 3 | 6 | 5 | 1 | 3 | .. | .. | .. | .. | .. | .. | .. |
| Septum Nasi, | 4 | 1 | 3 | .. | .. | .. | .. | .. | 4 | 3 | .. | 1 | .. | .. | .. | .. | .. | .. | .. |
| Sup. Maxilla, | 1 | 1 | .. | .. | .. | .. | .. | .. | 1 | .. | .. | .. | .. | 1 | 1 | .. | .. | .. | .. |
| Inf. Maxilla, | 13 | 7 | 6 | .. | .. | .. | 6 | 3 | 1 | 2 | 2 | 7 | 4 | 3 | 1 | 1 | .. | .. | .. |
| Clavicle, | 41 | 15 | 26 | 32 | 9 | .. | .. | 4 | 37 | 10 | 8 | 9 | 3 | 14 | 2 | 7 | 2 | .. | .. |
| Scapula, | 3 | 1 | 2 | .. | .. | .. | .. | .. | 3 | .. | .. | 2 | .. | .. | .. | .. | .. | 1 | 1 |
| Humerus, | 38 | 17 | 21 | 5 | 7 | 26 | 2 | 1 | 36 | 18 | 8 | 5 | 3 | 10 | 4 | 3 | 2 | 2 | .. |
| Radius, | 27 | 17 | 10 | 3 | 4 | 20 | .. | 2 | 25 | 12 | 9 | 7 | 5 | 6 | 2 | 2 | .. | .. | .. |
| Ulna, | 23 | 14 | 9 | 10 | 6 | 6 | .. | 2 | 20 | 10 | 8 | 8 | 5 | 4 | 1 | 1 | .. | .. | .. |
| Radius and Ulna, | 34 | 25 | 9 | 2 | 11 | 20 | .. | 2 | 31 | 23 | 18 | 5 | 3 | 5 | 3 | .. | .. | 1 | 1 |
| Femur, | 73 | 14 | 59 | 23 | 38 | 7 | 3 | 1 | 67 | 23 | 10 | 10 | 3 | 19 | 1 | 5 | .. | 6 | .. |
| Patella, | 7 | 1 | 6 | .. | .. | .. | .. | .. | 7 | 1 | .. | 2 | 1 | 4 | .. | 1 | .. | .. | .. |
| Tibia, | 19 | 14 | 5 | 3 | 10 | 6 | .. | 6 | 13 | 3 | 2 | 9 | 5 | 3 | 3 | 5 | 4 | .. | .. |
| Fibula, | 16 | 8 | 8 | 1 | 3 | 12 | 1 | 4 | 11 | 2 | .. | 4 | 2 | 6 | 4 | 2 | 2 | .. | .. |
| Tibia and Fibula, | 73 | 23 | 50 | 7 | 20 | 45 | 13 | 19 | 40 | 11 | 5 | 15 | 5 | 22 | 5 | 1 | .. | .. | .. |
| Carpal, | 1 | .. | 1 | .. | .. | .. | 1 | .. | .. | .. | .. | 1 | .. | .. | .. | .. | .. | .. | .. |
| Metacarpal, | 3 | 1 | 2 | 1 | .. | 2 | .. | 1 | 2 | 1 | .. | 2 | 1 | .. | .. | .. | .. | .. | .. |
| Phalanges, | 1 | .. | 1 | .. | .. | .. | .. | 1 | .. | 1 | .. | .. | .. | .. | .. | .. | .. | .. | .. |
| Ribs, | 4 | 2 | 2 | .. | .. | .. | .. | .. | 4 | .. | .. | 1 | .. | 3 | 2 | .. | .. | .. | .. |
| Vertebra, | 3 | .. | .. | .. | .. | .. | .. | .. | 3 | 2 | .. | .. | .. | .. | .. | 1 | .. | .. | .. |
| Pelvis, | 2 | .. | 2 | .. | .. | .. | .. | .. | 1 | .. | .. | .. | .. | 2 | .. | .. | .. | .. | .. |
| Amount, | 395 | 161 | 231 | 87 | 108 | 144 | 26 | 49 | 312 | 126 | 71 | 93 | 40 | 102 | 29 | 29 | 10 | 10 | 2 |

SUMMARY OF BONES OF SKULL FROM THE NOTES OF DR. HAMILTON, &c.

| Name of Bones, &c. | No. of Fractures. | Recovered. | Died. | Comp. & Commin. | Compound. | Simple. | To 15 yrs. N. | To 15 yrs. R. | 15 to 30 y. N. | 15 to 30 y. R. | 30 to 45 y. N | 30 to 45 y. R. | 45 to 60 y. N. | 45 to 60 y. R. | 60 to 82 y. N. | 60 to 82 y. R. |
|---|---|---|---|---|---|---|---|---|---|---|---|---|---|---|---|---|
| Os Occipital, | 2 | 1 | 1 | .. | 1 | 1 | 1 | 1 | .. | .. | 1 | .. | .. | .. | .. | .. |
| Os Parietal, | 18 | 13 | 5 | 7 | 8 | 3 | 12 | 11 | 3 | 1 | 3 | 1 | .. | .. | .. | .. |
| Os Frontis, | 9 | 5 | 4 | 3 | 5 | 1 | 4 | 2 | 2 | .. | 2 | 2 | .. | .. | 1 | 1 |
| Os Temporal, | 1 | 1 | .. | .. | .. | 1 | .. | .. | 1 | 1 | .. | .. | .. | .. | .. | .. |
| Occipital and Frontal, | 1 | 1 | .. | 1 | .. | .. | .. | .. | 1 | 1 | .. | .. | .. | .. | .. | .. |
| Os Frontis and Ethmoid, | 1 | .. | 1 | 1 | .. | .. | 1 | .. | .. | .. | .. | .. | .. | .. | .. | .. |
| Os Frontis, Ethmoid, Sphenoid and Temporal, | 1 | .. | 1 | .. | 1 | .. | .. | .. | .. | .. | 1 | .. | .. | .. | .. | .. |
| Amount, | 33 | 21 | 12 | 12 | 15 | 6 | 18 | 14 | 7 | 3 | 7 | 3 | .. | .. | 1 | 1 |
| Fractures of the Cranium at the N. Y. Hospital, by Dr. T. D. Lente, | 117 | 21 | 96 | .. | .. | .. | 13 | 4 | 64 | 11 | 39 | 6 | 9 | .. | 2 | .. |

Summary of Bones of Skull.—I have taken the liberty of forming a summary from the valuable article "Lente's Statistics of Fracture of the *Cranium*," published January last in the "New York Journal of Medicine and the Collateral Sciences." In those tables *one hundred and twenty-eight* cases are reported, of which the result in *three* cases was not given, and in *eight* others the age was wanting; on this account I have omitted these in my summary of his tables. These results differ from those obtained from Dr. Hamilton's notes, but the reader, if he examines the cases in both tables will perceive that those recorded by Dr. Lente are such as have been brought into the *New York Hospital* during the last twelve years, the result of *recent* injuries, and also that the proportion of those under *fifteen* years of age is very small compared to the other ages, while in Dr. Hamilton's notes more than one half occurred in the *first fifteen* years of life; also Dr. Hamilton's notes are *in part* composed of cases, which have come under his eye, after the lapse of several years from the time of the accident, and they cannot therefore so faithfully represent the average result of such injuries, since none but survivors are accounted for.

In a medico-legal point of view, the following suggestion of Dr. Lente, in the same article, is of value: "In no case did death follow the receipt of the injury until after the lapse of some hours, even in the most desperate cases; nor does it appear to be possible for an ordinary blow upon the head, producing fracture of the skull, to cause immediate death." "In a recent criminal trial of great interest, it will be recollected that, at one stage of the proceedings, it was much discussed whether a blow upon the head with an ordinary weapon capable of inflicting death, could produce this result instantaneously; many eminent surgeons were examined, and the general impression was that the thing was exceedingly improbable, if not impossible, and the question was thus decided." A suggestion, that the reader will see is equally applicable to the notes of the thirty-three cases by Dr. Hamilton.

In the March number of the same Journal, Stephen Smith, M. D., in an article entitled "Smith on Surgical Treatment of Epilepsy," exhibits one of the frequent results of fractures of the cranium, as illustrated also by Dr. Hamilton's tables. In twenty-seven cases of epilepsy reported by Smith, twenty-four were known to have resulted from fracture of the bones of the head.

The following additional remarks, although somewhat desultory, seem sufficiently pertinent:

Children, or for the sake of a convenient reference, I will say those of the *first period* in Dr. Hamilton's tables (the first fifteen years) show a large percentage of perfect results, in all cases of fractures.

*Ossa Nasi.*—Dr. Hamilton has observed that the accident called "fracture of the ossa nasi" is generally rather a displacement; the fracture being only at the serrated margins, where they unite with each other and with the neighboring bones.

It is a curious fact that only a small proportion of these patients apply to a surgeon for treatment. In the notes I find recorded fourteen cases, one of which died within a few days from other injuries, and seven others employed no surgeon, and have not therefore been admitted to these tables.

*Clavicle.*—It will be seen by the summary, that a large proportion of the fractures of this bone, are at the inner end of the outer $\frac{1}{3}$. Also that the number of perfect, after the first fifteen years of life is very small.

A great proportion of the fractures of carpal, metacarpal, phalangeal, tarsal and metatarsal, and also vertebral bones, occurring in Dr. Hamilton's practice, or coming under his observation, have not been recorded. The tables are not, therefore, a correct representation of their relative frequency.

The New York Journal, before alluded to, vol. iii, 1840, speaks in very high terms of "Remarks on Fractures, by A. L. Pierson, M. D., of Salem, Mass.," which contains not only remarks, but also tables "drawn from the records of the Mass. General Hospital," "of all the cases of fracture ever treated in that institution." Also the Boston Med. and Surg. Jour., Aug., 1840, makes honorable mention of those tables. I regret that I have not had the opportunity of examining them.

In the same Journal, vol. iv., page 473, is a short article entitled "Cases of Fracture in the General Hospital of Hamburgh, during the year 1838, by Fricke." "The whole number treated was *seventy-two*. Of these, forty-two were cured, ten died, and twenty remained under treatment." Between 10 and 30 years there were twenty fractures; from 30 to 50 years, twenty-four fractures; 50 to 70 years, twenty-two fractures; 70 to 90 years, six fractures. Also the tibia and fibula were broken *twenty-two* times, the femur *seventeen* times, os brachii *seven*, and the clavicle *three* times—the *shortest term of treatment* was for the os nasi, *nineteen days*; and the *longest* for the neck of os femoris, *one hundred and thirty-nine days.*

The author states that of the seventy-two, six remained from the last year; of the sixty-six who entered, fifteen were females.

Lente, N. Y. Jour., vol. iii, page 167, gives a statement of 1548 fractures, of which 1366 were males, and 182 females. I have not been able to determine the relative proportion in which fractures occur of the sexes: these, being the only statements I have been able to find on this point.

The only records I have found of the number of fractures drawn from a body of men, were the books of the Auburn State Prison. They show that of *five hundred and eighty* convicts admitted, *one hundred and twenty* reported at time of admission, ancient fractures.

The subject of *results*, which occupies the most prominent place in Dr. Hamilton's tables, is not considered in Fricke's table, nor am I informed that it constitutes a point in Pierson's tables, or in any others hitherto constructed. I am at least quite certain that by no one else, have the results been determined by measurements.

"Lente's Statistics of Fractures" published in the September number of the N. Y. Journal, give the "age, sex, season, and seat of fracture, &c.," in those fractures occurring in the N. Y. Hospital for the last twelve years, but in his tables, he makes no mention of the amount of shortening in the different fractures. To show the relative frequency of fractures of the different bones, he institutes "a comparison with the statistics of Malgaigne, at the Hotel Dieu of Paris, Lonsdale, at the Middlesex Hospital of London," and Norris, at Pennsylvania Hospital of Philadelphia; from which, as well as from Fricke, at the General Hospital at Hamburgh, and Dr. Hamilton's notes, I shall make the following abridgement by way of comparison.

| NAME OF BONES. | N. Y. Hospital. | Pennsylvania Hospital. | Hotel Dieu. | Middlesex Hospital. | General Hospital at Hamburgh. | Dr. Hamilton. |
|---|---|---|---|---|---|---|
| Thigh, | 280 | 134 | 308 | 181 | 17 | 73 |
| Tibia and Fibula, | 442 | | 515 | 197 | 22 | 73 |
| Tibia, | 45 | 293 | 29 | 41 | 5 | 19 |
| Fibula, | 92 | | 108 | 51 | 4 | 16 |
| Arm, | 161 | | 310 | 118 | 7 | 38 |
| Radius and Ulna, | 90 | | 107 | 93 | 2 | 34 |
| Ulna, | 36 | 227 | 29 | 64 | .. | 23 |
| Radius, | 143 | | 160 | 197 | 1 | 27 |
| Clavicle, | 158 | 84 | 225 | 273 | 3 | 41 |
| Inf. Maxilla, | 65 | 19 | 27 | 32 | 1 | 13 |
| Pelvis, | 23 | 6 | 7 | 7 | 3 | 2 |
| Patella, | 30 | 16 | 45 | 38 | 1 | 7 |
| Scapula, | 17 | 10 | 4 | 18 | .. | 3 |
| Sternum, | 12 | 5 | 1 | 2 | 1 | 1 |
| Skull, | 128 | 46 | 53 | 48 | .. | 33 |
| Amount, | 1722 | 840 | 1939 | 1392 | 67 | 403 |

It is thus by comparison of the cases occurring both here and elsewhere, that I have endeavored to arrive at the general truths, which can only be obtained from facts collected from various sources. It is by comparison of the *results* in the tables first published by Dr. Hamilton, with those I have compiled from his notes, and also by comparing both with other tables, that I have sought to prove their accuracy as statistical records. There will be seen a striking similarity in the average results between the tables in the case of almost every bone; the relative frequency of fracture being nearly identical, as stated by the different writers. It is true the statistics of Middlesex Hospital present a much larger proportion of fractures of the superior extremities than the other tables; but when it is ascertained that the number of beds in this Hospital is limited, and that a large proportion of those treated are "out-door patients," this disagreement will find a sufficient explanation.

